# Supplementary material for: Microglia permit climbing fiber elimination by promoting GABAergic inhibition in the developing cerebellum
Source: Nat Commun. 2018 Jul 19;9:2830. doi: 10.1038/s41467-018-05100-z (PMC6053401; doi:10.1038/s41467-018-05100-z)
Supplement: Supplementary file 1 — Supplementary Information [file 41467_2018_5100_MOESM1_ESM.pdf]

Microglia permit climbing fiber elimination by promoting GABAergic inhibition in the developing cerebellum

Nakayama et al.

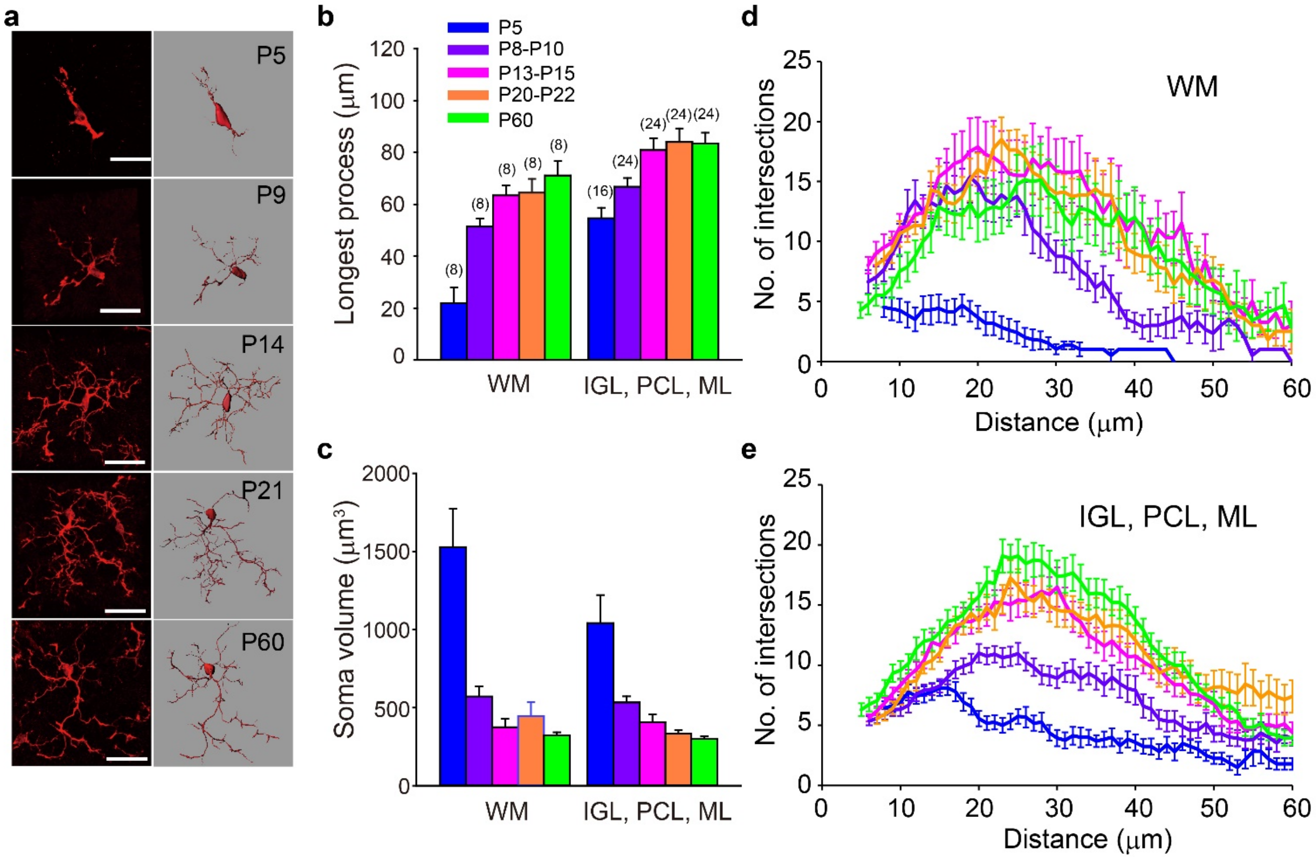

Supplementary Figure 1. Postnatal changes in the morphology of microglia.

(**a**) Iba1-positive microglial images (left) and reconstructions (right) at P5, P9, P14, P21, and P60. Scale bars, 30 μm. (**b**) The average length of the longest process at P5 (blue), P8–P10 (purple), P13–P15 (pink), P20–P22 (orange) and P60 (green) in the WM (left) and the gray matter except the EGL (IGL, PCL, ML; right). Number of analyzed cells are presented above individual bars. (**c**) Similar to **b**, showing data for the average volume of microglia soma. Numbers of analyzed microglia are the same as in **b**. (**d**) Sholl analysis for microglial processes at P5 (blue), P8–P10 (purple), P13–P15 (pink), P20–P22 (orange) and P60 (green) in the white matter. Eight microglia were analyzed at each postnatal period. (**e**) Similar to **d**, but data are for microglia in the gray matter except the EGL. Sixteen to twenty-four microglia were analyzed at each postnatal period. Data are presented as mean ± SEM.

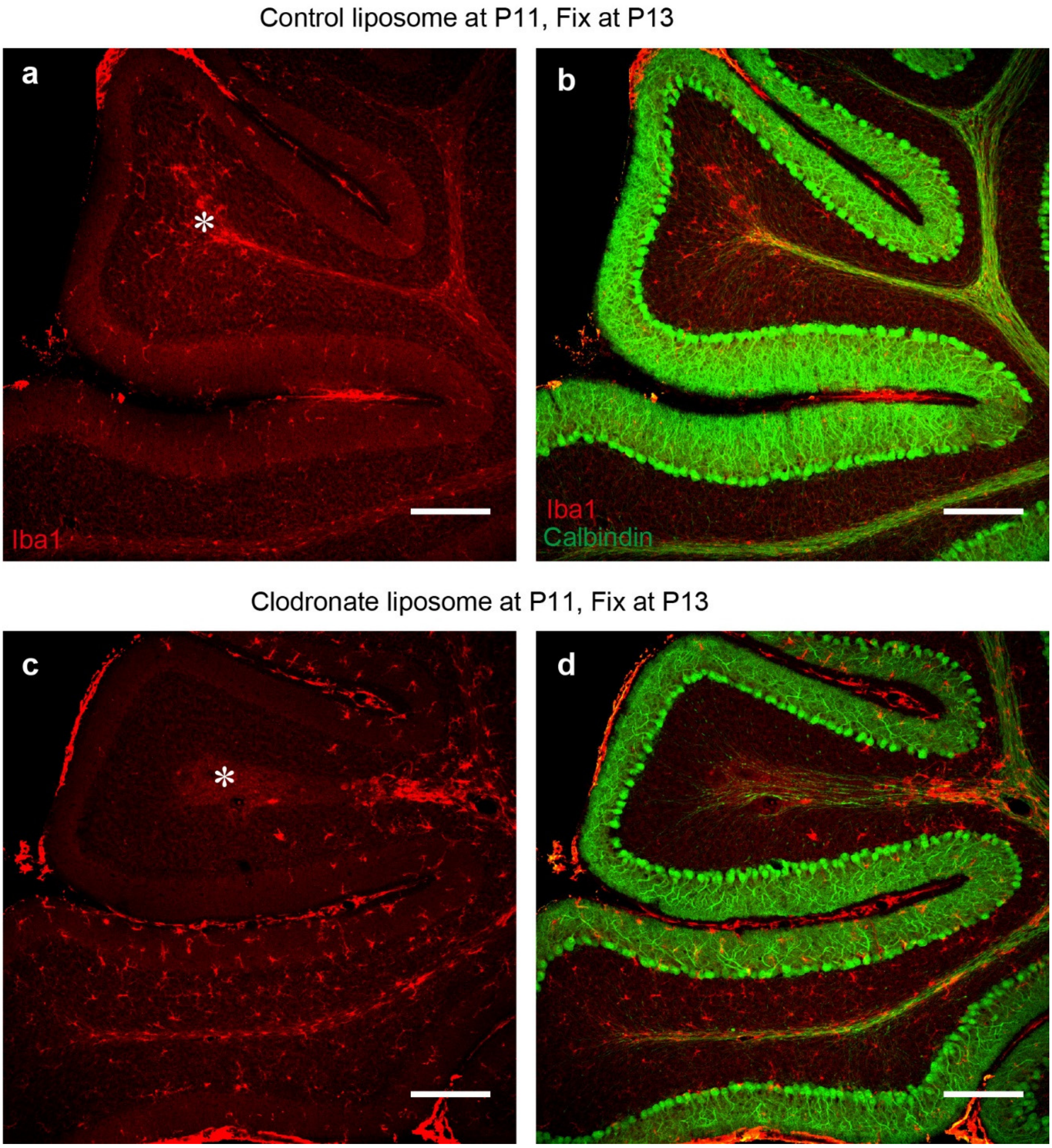

Supplementary Figure 2. Local injection of liposomal clodronate at P11 deletes microglia.

Control liposomes (**a**,**b**) or liposomal clodronate (**c**,**d**) were injected into lobule VIII of cerebellar vermis of C57BL/6 at P11. The mice were fixed at P13 and immunostained for Iba1 and calbindin. Iba1-positive microglia are present in the control liposome-injected lobule (asterisk in **a**) but disappeared in the liposomal clodronate-injected lobule (asterisk in **c**). Distribution of calbindin-labeled PCs is largely unchanged by the liposome injection (**b**, **d**). Scale bars, 200 μm.

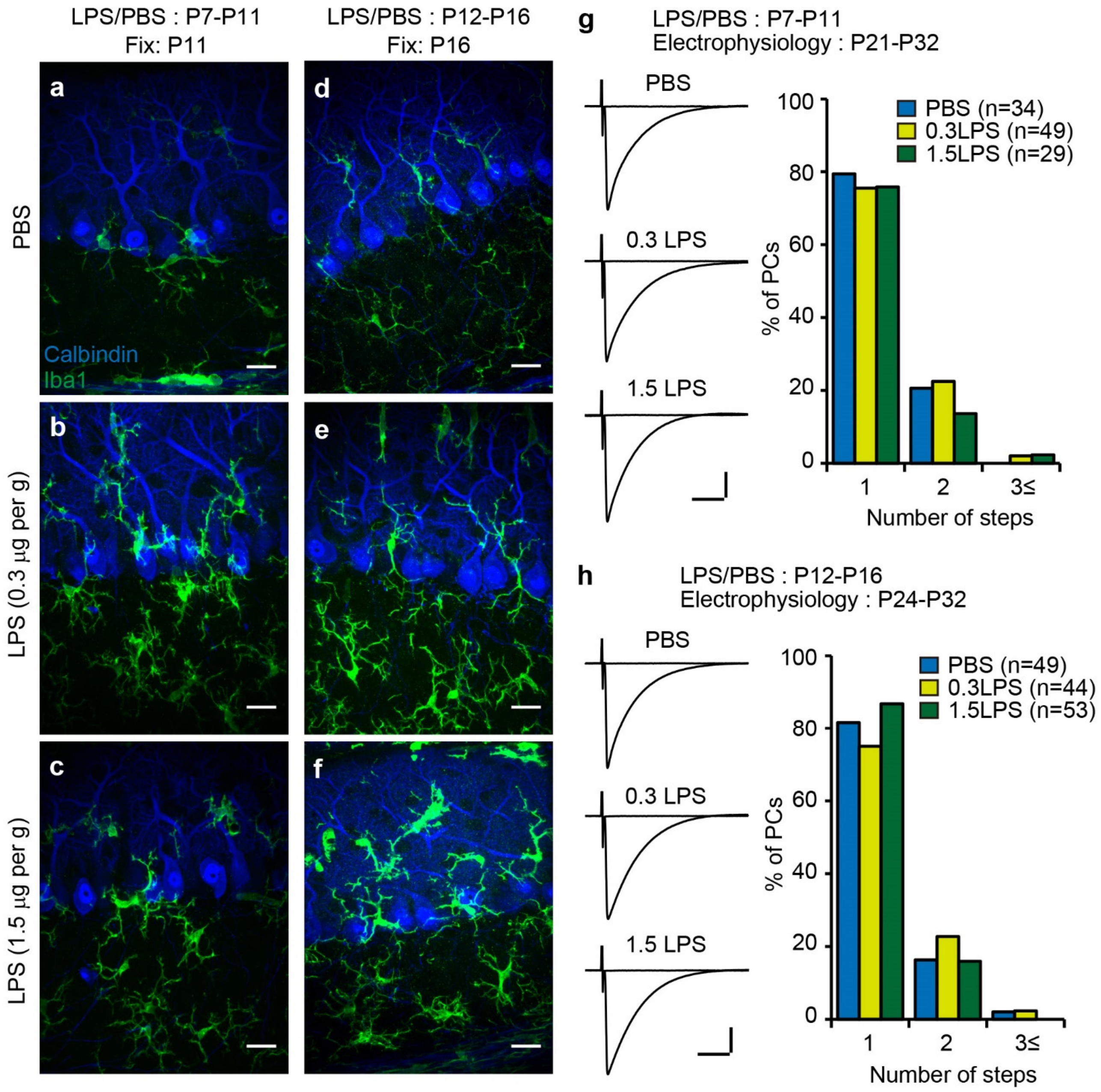

Supplementary Figure 3. Synapse elimination is normal in LPS-treated mice.

LPS (0.3 μg per g or 1.5 μg per g of body weight) or PBS was intraperitoneally injected into C57BL/6 mice once per day at P7–P11 (**a–c**,**g**) or P12–P16 (**d–f**,**h**). (**a–f**) Immunostaining for calbindin (blue) and Iba1 (green). Mice were fixed on the last day of the LPS or PBS injection (P11 in **a–c**, P16 in **d–e**). Immunofluorescence of Iba1 is strongly enhanced and microglial processes are thick in LPS-treated mice, suggesting that microglia respond to LPS. Scale bars, 20 μm. (**g**,**h**) (Left) CF-EPSCs recorded from a PBS- (upper), a 0.3 LPS- (middle) or a 1.5 LPS- (bottom) treated mouse; Vh = −10mV. Scale bars, 500 pA and 10 ms. (right) Frequency distribution histograms showing the number of discrete CF-EPSC steps at P21–P32. There was no significant difference among the three groups (**g**, $p = 0.886$; **h**, $p = 0.318$, Kruskal-Wallis test). Each dataset was obtained from 2–3 mice.

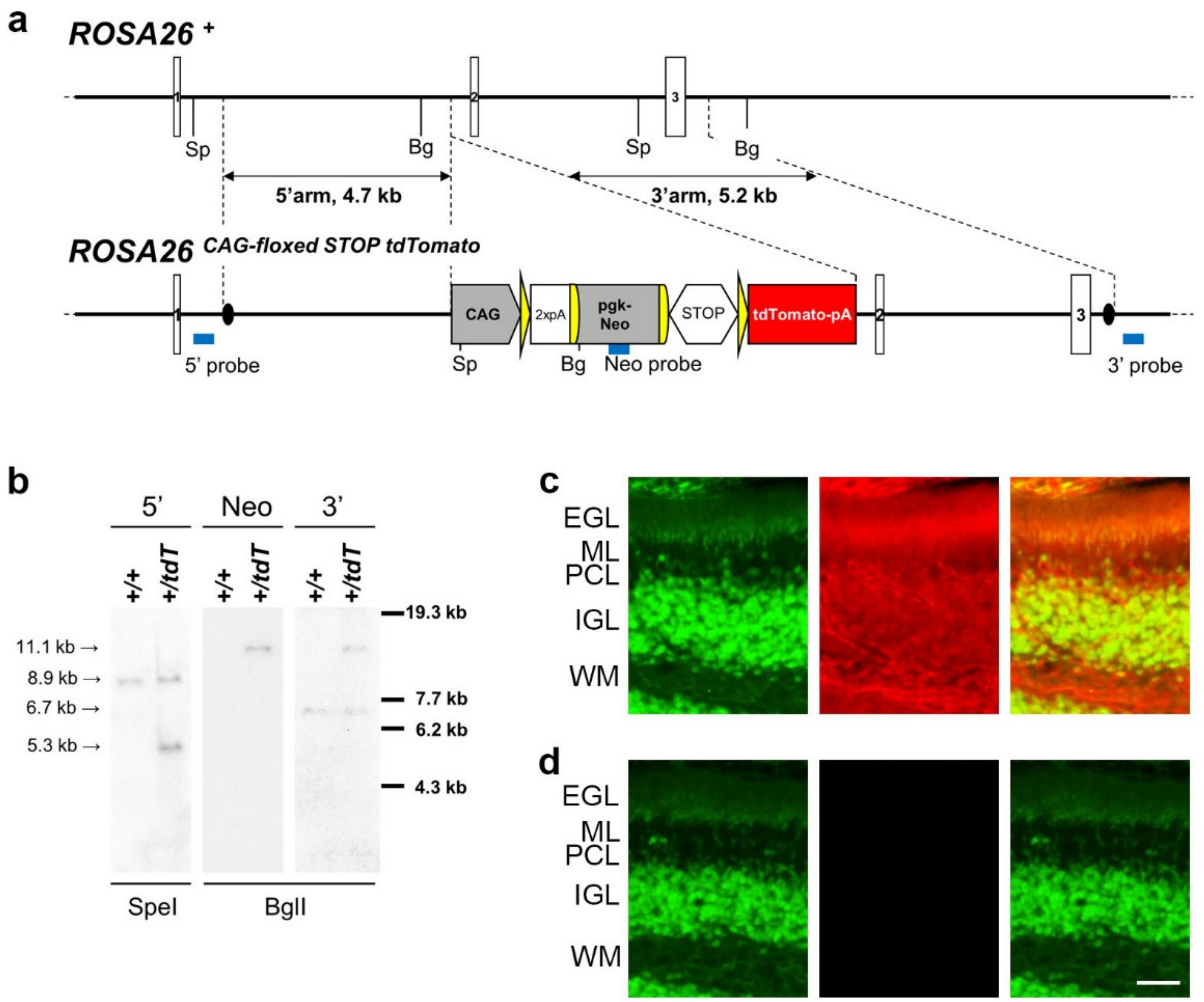

Supplementary Figure 4. Generation of the CAG-floxed STOP tdTomato reporter line.
(**a**) Schematic representations of *ROSA26* genomic DNA and the targeted genome. The open boxes indicate the noncoding exons. The filled circles in the targeted allele delineate the 5′ and 3′ termini of the targeting vector. The vector was constructed to insert a CAG promoter (CAG), floxed polyadenylation signal (pA)-Neo-STOP cassette[1] and tdTomato gene into intron 1 of the *ROSA26* locus. The blue bars indicate the probes for Southern blot analysis; loxP and frt sequences are indicated by triangles and semicircles, respectively. Bg, *Bgl*I; Sp, *Spe*I. (**b**) Southern blot analysis for genomic DNAs from wild-type (+/+) and targeted (+/tdT) mice. Left, *Spe*I-digested genomic DNA hybridized with 5′ probe; middle and right, *Bgl*I-digested DNA hybridized with Neo or 3′ probe, respectively. (**c, d**) Immunostaining for NeuN (green) and tdTomato fluorescence (red) in lobules IV–V in the vermis of the cerebellum at P7. (**c**) Fluorescence images in a CAG-tdTomato (ΔSTOP) mouse established by germline Cre recombination. (**d**) Fluorescence images in a CAG-floxed STOP tdTomato mouse without the Cre gene. A scale bar, 100 μm.

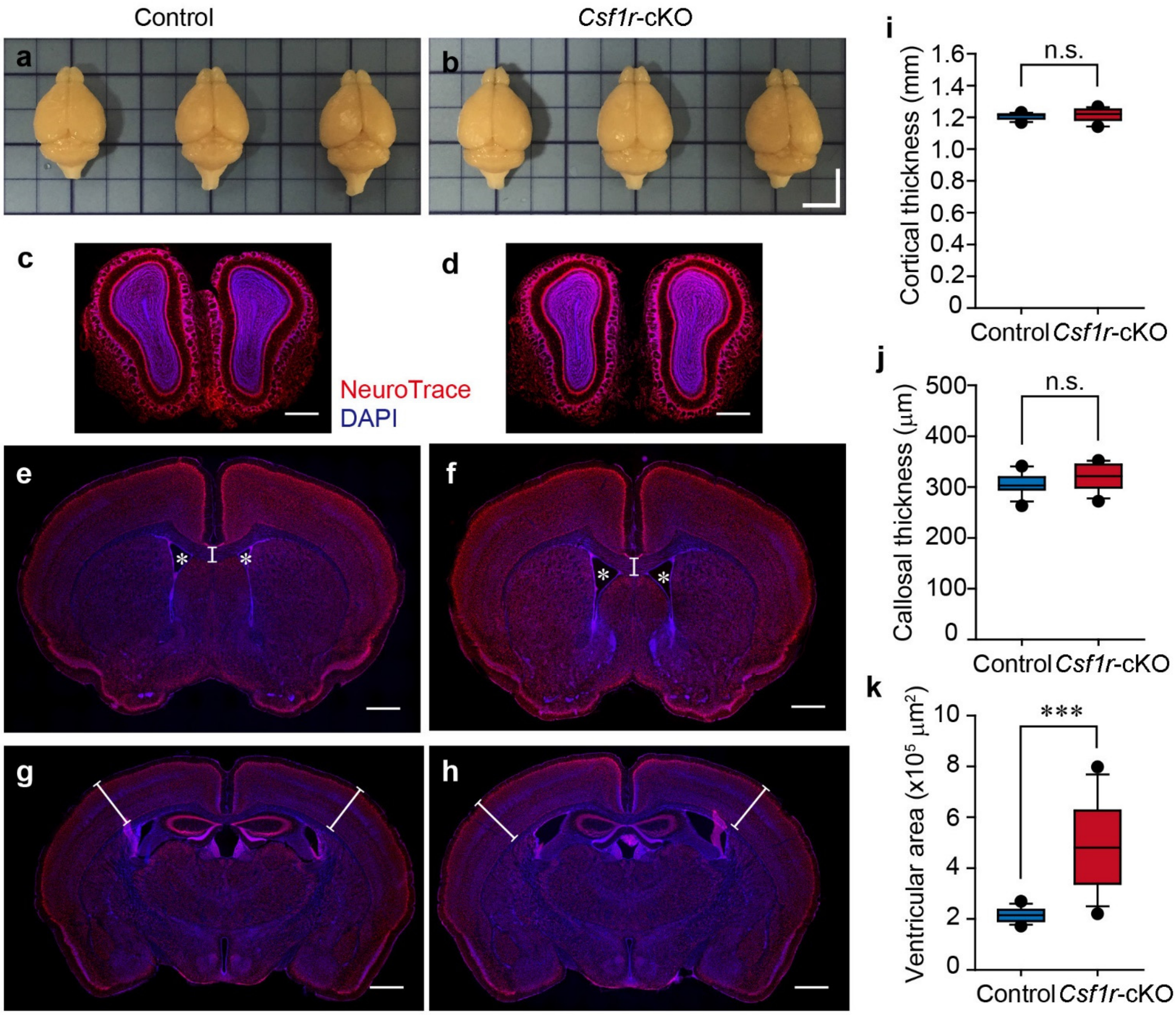

Supplementary Figure 5. Gross brain architecture in *Csf1r*-cKO mice.

(**a**,**b**) Dorsal view of whole brains of control (**a**) and *Csfr1*-cKO mice (**b**) at P35. The brain sizes were largely not different between control mice and *Csf1r*-cKO mice. Scale bars, 5 mm and 5 mm. (**c**–**h**) Coronal brain slices at about 4.3 mm (**c**, **d**), 0.5 mm (**e**, **f**) and −1.2 mm (**g**, **h**) rostral to the Bregma, sampled from control (**c**, **e**, **g**) or *Csf1r*-cKO (**d**, **f**, **h**) mice. Slices were stained with Neuro Trace 530/615 (red) and DAPI (blue). Scale bars, 500 µm. (**i**, **j**) Thickness of the cerebral cortex (**i**) or the corpus callosum (**j**) measured at the white lines in **g**,**h** and **e**,**f**, respectively. There were no differences between control and *Csf1r*-cKO mice (**i**, $p = 0.371$; **j**, $p = 0.199$). (**k**) Ventricular area measured at the asterisk-denoted region (in **e**,**f**) in *Csf1r*-cKO mice was significantly enlarged ($p < 0.001$). The box plots were obtained from 12 brain slices from three control mice and 12 brain slices from three *Csf1r*-cKO mice. n.s., $p > 0.05$.

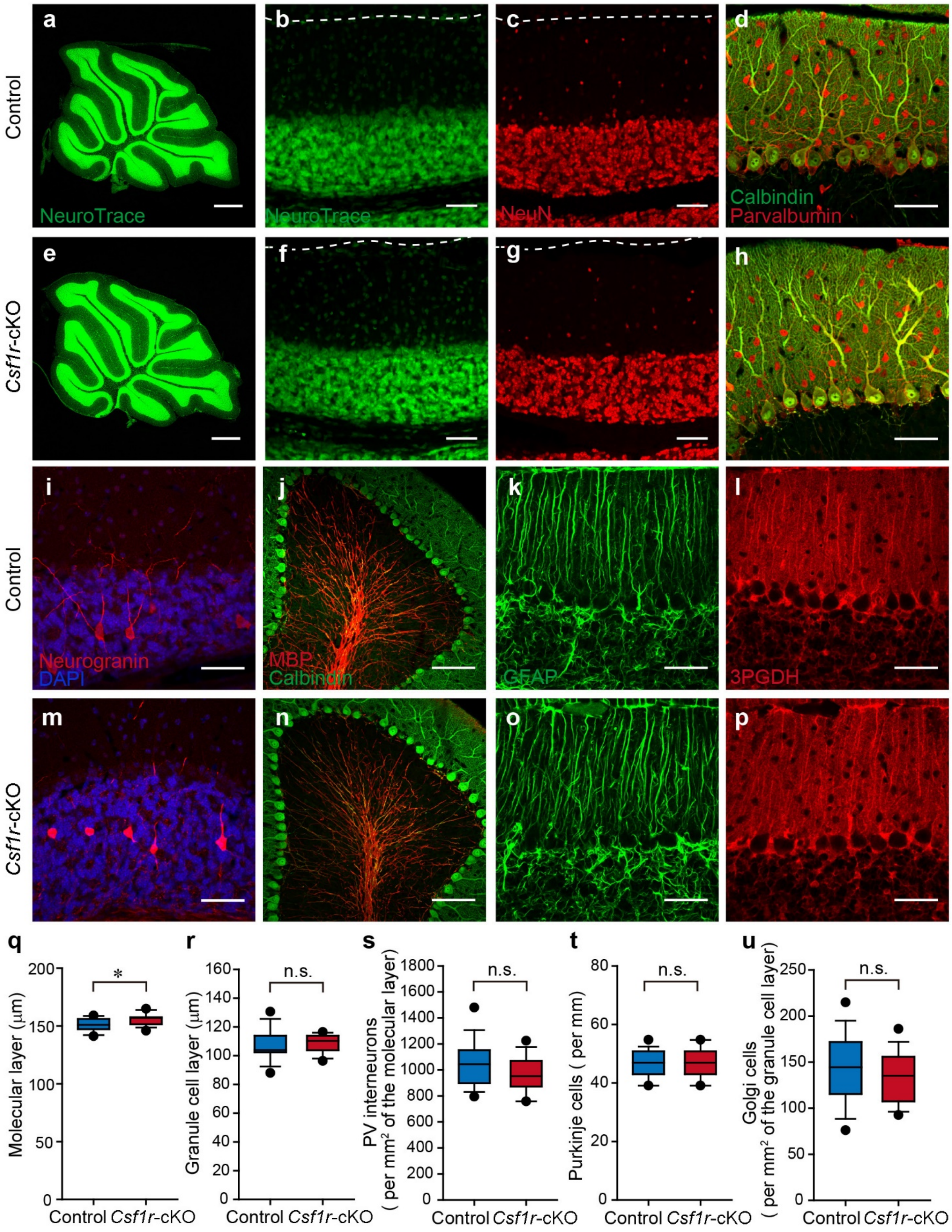

Supplementary Figure 6. Morphological characterization of the cerebellar cortex in *Csf1r*-cKO mice

(a,e) Foliation and lobular structure of vermis in control (a) or *Csf1r*-cKO (e) mice. Slices were stained with Neuro Trace for Nissl staining. (b,f) Higher magnification images of Neuro Trace staining in control (b) or *Csf1r*-cKO (f) mice. (c,g) Similar to b, f, but images are of immunostaining for NeuN as a marker for granule cells. (d,h) Merged images for parvalbumin (PV, red), a marker for inhibitory interneurons, and calbindin (green), a marker for Purkinje

cells. (**i,m**) Merged images for neurogranin (red), a marker of Golgi cells, and DAPI (blue). (**j,n**) Merged images for MBP (red), a marker for myelinating oligodendrocytes, and calbindin (green). (**k,o**) Immunostaining for GFAP, a marker for astrocytes. (**l,p**) Immunostaining for 3PGDH, a marker for Bergmann glia. (**q,r**). Box plots showing the thickness of the ML (**q**) and the GCL (**r**). Thickness of the GCL is normal (**r**, $p = 0.600$), but the ML in *Csf1r*-cKO mice is slightly thicker than in control mice (**q**, $p = 0.0267$, *t*-test). (**s–u**) Boxplots showing densities of PV-interneurons (**s**), PCs (**t**) and Golgi cells (**u**). All indexes show no difference between control and *Csf1r*-cKO mice (**s**: $p = 0.348$; **t**: $p = 0.884$; **u**: $p = 0.489$, *t*-test). Data for the box plots were from 15 images taken from three control and three *Csf1r*-cKO mice. All images, except for **a** and **e**, were taken from lobules IV–V of P22–P35 mice. Scale bars, 500 μm (**a,e**), 50 μm (**b–d,f–i,k-m,o,p**) and 100 μm (**j,n**). *, $p < 0.05$; n.s., $p > 0.05$.

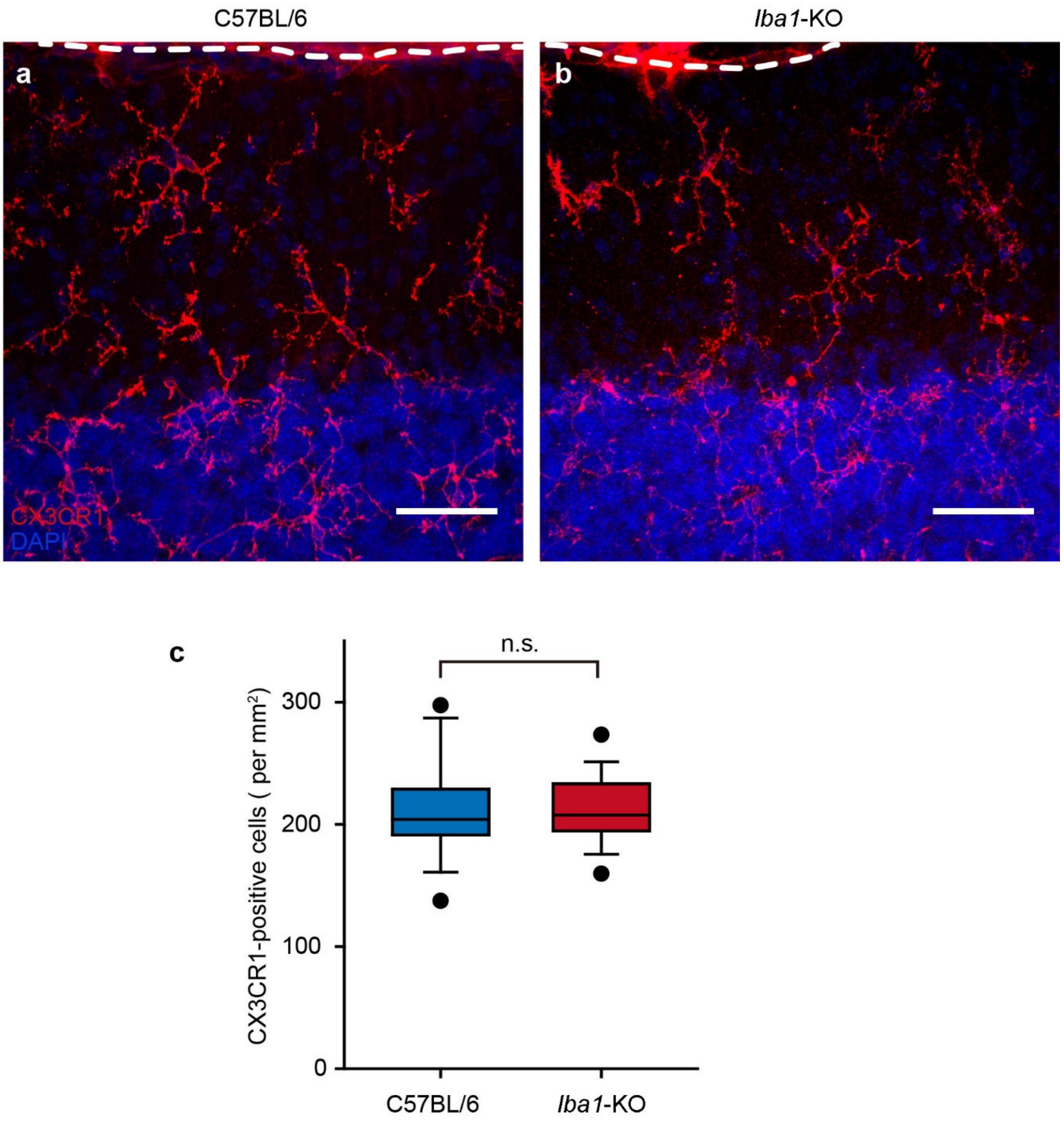

Supplementary Figure 7. CX3CR1-positive microglia in the *Iba1*-KO mouse cerebellum.
(**a**,**b**) Immunostaining for CX3CR1 (red), a marker for microglia, and DAPI (blue) in C57BL/6 (**a**) or a *Iba1*-KO (**b**) cerebellum. Images were taken from lobules IV–V of the cerebellar vermis at P22. Scale bars, 50 μm. (**c**) A box plot showing the density of CX3CR1-positive microglia per mm^2. The density is unchanged in *Iba1*-KO mice ($p = 0.947$, *t*-test). Data for the box plots were from 15 images taken from three control and three *Iba1*-KO mice. n.s., $p > 0.05$.

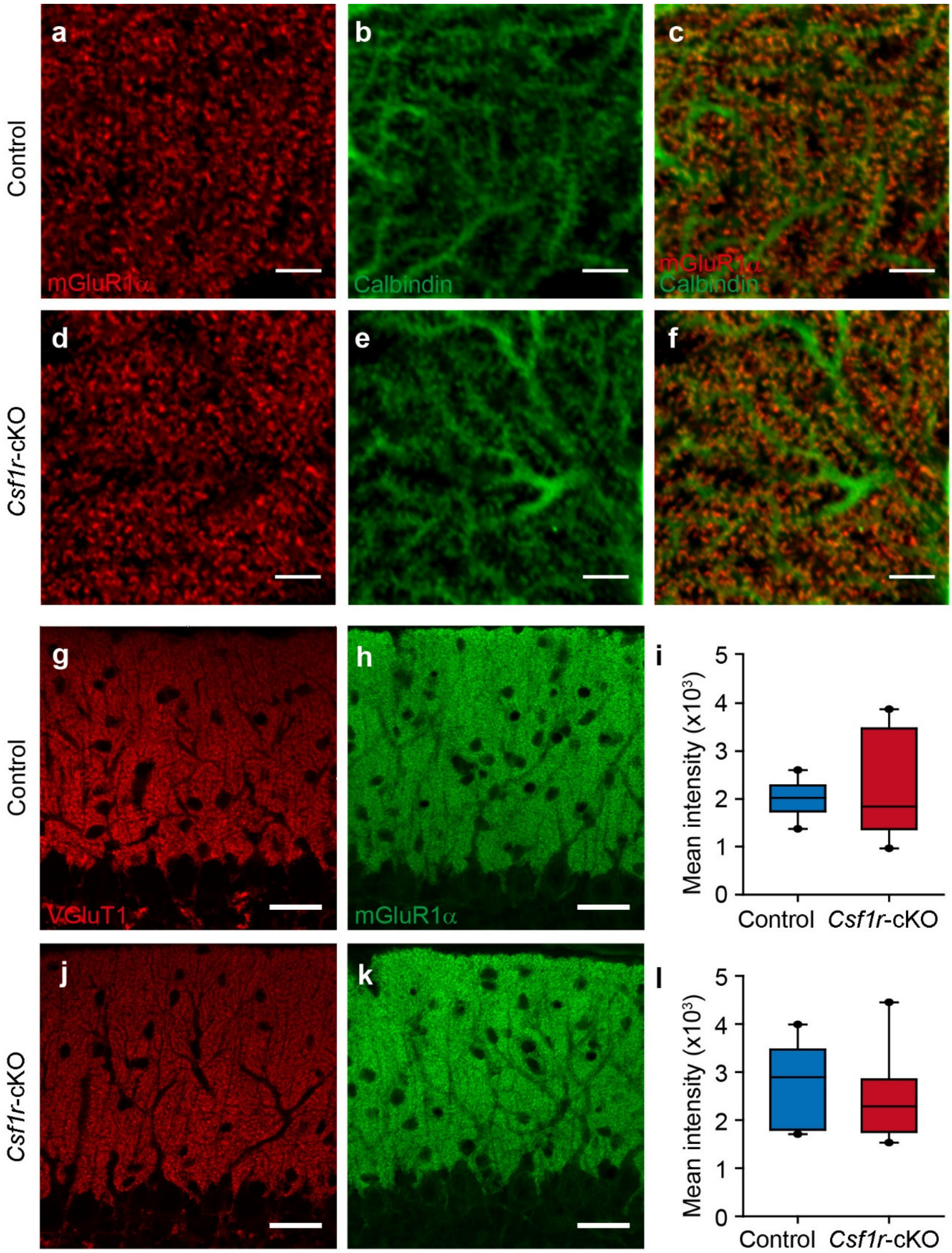

Supplementary Figure 8. Immunostaining for mGluR1α and VGluT1.
(**a–f**) High magnification images of immunostaining for mGluR1α (red, **a**,**d**), calbindin (green, **b**,**e**) and merged images (**c**,**f**) in the ML in a control (**a–c**) or *Csf1r*-cKO (**d–f**) mouse. The mGluR1α puncta are on PC spines in both control and *Csf1r*-cKO mice (**c**,**f**). (**g**,**h**,**j**,**k**) Low magnification images of immunostaining for VGluT1 (**g**,**j**) and mGluR1α. (**h**,**k**). (**i**,**l**) Mean fluorescence intensities of VGluT1 (**i**) and mGluR1α (**l**) within the ML. There was no significant difference between control and *Csf1r*-cKO mice (VGluT1: $p = 0.612$; mGluR1α: $p = 0.549$, *t*-test). Data for the box plots were from 8 images taken from four control and three *Csf1r*-cKO mice. Images were taken from lobules IV–V of the cerebellar vermis at P16–P18. Scale bars, 5 μm (**a–f**) and 30 μm (**g**,**h**,**j**,**k**).

Supplementary Table 1. Kinetics of CF-EPSCs in clodronate-treated mice.

	Amplitude (pA)	10-90% Rise time (ms)	Decay time constant (ms)	Paired-pulse ratio	n
Control	2413 ± 152	0.51 ± 0.02	10.70 ± 0.44	0.70 ± 0.01	34
Clodronate	2627 ± 146	0.46 ± 0.02	9.88 ± 0.42	0.69 ± 0.02	38
p	0.359	0.101	0.217	0.431	

The decay time constant of CF-EPSCs was calculated by fitting the decaying phase of the EPSC with a single exponential. The paired-pulse ratio of CF-EPSCs in response to a double stimulus with an interval of 50 ms was defined as the percent of the second EPSC amplitude relative to the first.

Supplementary Table 2. Kinetics of CF-EPSCs in *Csf1r*-cKO mice.

	Amplitude (pA)	10-90% Rise time (ms)	Decay time constant (ms)	Paired-pulse ratio	n
Control	2124 ± 84	0.44 ± 0.01	7.09 ± 0.26	0.71 ± 0.01	54
Csf1r-cKO	2506 ± 97	0.40 ± 0.01	6.56 ± 0.18	0.69 ± 0.01	58
p	0.002	0.001	0.062	0.072	
	Disparity ratio		Disparity index		n
Control	0.88 ± 0.11		0.29 ± 0.06		14
Csf1r-cKO	0.92 ± 0.06		0.23 ± 0.04		35
p	0.939		0.662		

The decay time constant of CF-EPSCs was obtained by fitting the decaying phase of the EPSC with a single exponential. The paired-pulse ratio of CF-EPSCs in response to a double stimulus with an interval of 50 ms was defined as the percent of the second EPSC amplitude relative to the first. The disparity ratio and disparity index were defined using the following formula[2]:

$$Disparity\ ratio = \frac{1}{N-1}\left(\frac{A_1}{A_N} + \frac{A_2}{A_N} + \cdots + \frac{A_{N-1}}{A_N}\right)$$

$$Disparity\ index = \frac{1}{\mu}\sqrt{\frac{1}{N-1}\sum_{i=1}^{N}(A_i - \mu)^2}$$

$$\mu = \frac{1}{N}\sum_{i=1}^{N} A_i$$

N ($N \geq 2$) is the number of CFs innervating a given PC. Each $A_1, A_2, \ldots, A_N$ is the amplitude of the CF-EPSC numbered in order. A_1 and A_N are the smallest and largest CF-EPSC amplitudes, respectively.

Supplementary References

1. Buch T*, et al.* A Cre-inducible diphtheria toxin receptor mediates cell lineage ablation after toxin administration. *Nature methods* 2, 419-426 (2005).

2. Hashimoto K, Kano M. Functional differentiation of multiple climbing fiber inputs during synapse elimination in the developing cerebellum. *Neuron* 38, 785-796 (2003).
